# Knowledge and Confidence Among Five Cohorts of Faculty Learners in a Point of Care Ultrasound (POCUS) Program: Factors Defining Learner Success 

**DOI:** 10.24908/pocus.v9i2.17640

**Published:** 2024-11-15

**Authors:** Michael Janjigian, Anne Dembitzer, Isaac Holmes, Caroline Srisarajivakul­klein, Khemraj Hardowar, Harald Sauthoff

**Affiliations:** 1 Department of Medicine, New York University Grossman School of Medicine, NYC Health & Hospitals/Bellevue NY USA (; 2 Department of Medicine, New York University Grossman School of Medicine, NY Harbor Healthcare System New York City, NY USA; 3 Department of Medicine, New York University Grossman School of Medicine, NYU Langone Health New York City, NY USA; 4 Department of Medicine, Westchester Medical Center New York City, NY USA

## Abstract

**Background: **The availability of faculty proficient in point of care ultrasound (POCUS) has emerged as a barrier to the ongoing expansion of POCUS across the field of internal medicine. We sought to examine the faculty graduates of our institutional POCUS program to identify characteristics associated with long-term proficiency to inform curricula and guide institutional support. **Methods:** We emailed a test and survey to the 89 faculty graduates of the annual POCUS course we have run from 2018-2023. **Results:** Of the 46 participants (52%) who completed the test and survey, the overall median test score was 72%. Graduates were most confident with image acquisition of the lung, and were most likely to use ultrasound to evaluate ascites and dyspnea. All 11 participants reporting completion of an image portfolio were actively teaching POCUS, whereas only 54% of non-completers were teaching. Portfolio completers scored higher on the test compared to non-completers (median 92% and 68%, respectively, p <0.01) and were more confident in image acquisition and interpretation (p<0.001). **Conclusions:** In this long-term, single-institutional study of faculty graduates of an annual POCUS program, those who reported completing an image portfolio scored significantly higher on a knowledge test, reported higher confidence with image acquisition and interpretation, and reported using and teaching POCUS more frequently compared to graduates who did not complete the portfolio. POCUS education programs should be designed to foster continuous scanning practice and image portfolio completion.

## Background

Point of care ultrasound (POCUS) has expanded rapidly across the undergraduate medical education (UME) and graduate medical educational (GME) landscapes and in the field of internal medicine. Multiple national medical associations currently support POCUS training for internal medicine residents and faculty, including the Alliance of Academic Internal Medicine, the American College of Physicians, the American Medical Association, and the Society of Hospital Medicine 1, 2, 3, 4, 5. Ultrasound education now begins at the earliest stages of medical training, with seventy-two percent of medical schools offering formal POCUS curricula 6. This expectation extends through a physician’s graduate training, with residents in multiple specialties, including internal medicine, expecting ongoing training in POCUS 6, 7, 8. Meeting these expectations requires academic centers to develop systems of growth and sustainability for POCUS education at all levels of training. A clear barrier to the growth of POCUS in internal medicine is the availability of faculty proficient in POCUS 9, 10. The lack of required training in residency has led faculty to rely on a network of local, regional, and national POCUS courses for training. There is a paucity of data regarding how effectively faculty learn POCUS. Numerous studies have demonstrated that faculty participants of a POCUS course learn the essential knowledge and skill components 11, 12. However, the majority of these graduates do not go on to achieve independent practice 11, 13, 14, 15. For example, a recent study found that of 102 hospitalists engaged in a POCUS curriculum, only 3 achieved independent practice 16. 

There is no consensus on effective practices to improve the rate of faculty independence in POCUS. Our group published outcomes for the 2018 cohort which demonstrated retention of hands-on skills and a small drop in score on the knowledge test one-year after course completion 17. The strongest behavioral correlate of hands-on skill retention at one-year was the practice of clip uploading followed by attendance at hands-on teaching sessions and attendance at monthly POCUS conferences. The approach of advancing faculty to become proficient in POCUS at Massachusetts General Hospital (MGH) follows a similar model as used at our institution by beginning with an intensive course (“bolus” phase) and supporting faculty during the longitudinal period (“drip” phase) 18. Their progress report describes the successful training of five teaching faculty who have become credentialed in POCUS at MGH through an intuitive framework for senior faculty development in POCUS. Studies of facilitators and barriers to faculty adoption of POCUS emphasize qualitative individual or environmental factors contributing to success in adopting POCUS 14, 19, 20. There remain gaps in our understanding as to why certain faculty taking the same course progress to independent practice while others may never pick up a probe again. We sought to elucidate participant characteristics and patterns of use that are associated with POCUS proficiency through surveying faculty graduates of our institutional Integrated Sonography Course at NYU (I-ScaN).

## Methods

I-ScaN Program Setting and Participants

The NYU Grossman School of Medicine academic system spans four teaching hospitals; NYU Langone Health (Tisch/Kimmel and Brooklyn campuses), Health + Hospitals/Bellevue, and the VA New York Harbor Health Care System Margaret Cochran Corbin Campus. Potential participants were identified through recommendation by divisional leadership at each site, targeting outpatient and inpatient physicians and Advanced Practice Providers (APPs). Additional participants were identified through self-referral from faculty, fellows and APPs in other divisions and departments. From April 2018 until June 1, 2023, 89 faculty learners had completed the course. 

I-ScaN Program Description

Beginning in 2018, I-ScaN has been offered at our institution, and with modifications made during the COVID pandemic 17, 21. The course starts with a one-month self-study period where participants are referred to relevant chapters from a POCUS textbook (22), selected articles, and online videos. The course is modeled on the American College of Chest Physicians Critical Care Ultrasound course 12, typically offered over two days. Systems covered in the course include cardiac (five standard views), lungs/pleura, abdomen (kidneys, bladder and aorta), and leg vasculature. Each system is taught with a didactic lecture reviewing theoretical concepts, an interactive image-based review of normal and abnormal findings, and hands-on training on a human model with a faculty to learner ratio of no more than 1:3. The longitudinal phase consisted of monthly conferences, directly supervised hands-on scanning sessions, and self-directed practice with clip uploads with expert review. Beginning during the COVID pandemic (2020), the monthly conferences were no longer offered while the directly supervised scanning sessions were continued at each clinical site.

Participants are encouraged to complete a comprehensive image portfolio identical to that required by the SHM POCUS Certificate of Completion 22. Upon satisfactory review of the portfolio by a local expert (HS or MJ), the participant is granted a Certificate of Completion of the I-ScaN program which is sent to the participant and their respective Chief of Medicine. 

I-ScaN Program Evaluation

On September 11, 2023, all 89 faculty graduates of the course between 2018-2023 were emailed a link and a weekly reminder via the Qualtrics Survey platform (Provo, Utah) to a 26-item knowledge test. The test was nearly identical to that previously administered for the initial course along with a survey assessing confidence in image acquisition and interpretation, integration of POCUS into clinical practice, facilitators and barriers to learning POCUS and demographic information (S1). The test assessed basic ultrasound concepts, image interpretation and clinical integration (S2). 

The I-ScaN program qualified as a quality improvement project by the NYU Grossman School of Medicine’s Institutional Review Board criteria using a self-certification process to ensure the data were not collected for research purposes. The primary goal of the project was to assess and improve educational performance of the I-ScaN program.

Statistics

We performed a single cross-sectional survey of faculty who had completed our program as one of five cohorts from 2018-2023. Knowledge scores were derived from the 26-item test, and confidence levels in acquiring and interpreting POCUS images were aggregated from the 10 specific survey items. Our analysis focused on two distinct groups: those who reported completing a portfolio versus those who did not, and individuals identified as having a high likelihood of utilizing POCUS for assessing dyspnea. We utilized the Wilcoxon rank sum test to assess the differences between these groups regarding their knowledge and confidence scores. The ANOVA test was used to assess for interaction effects between cohort grouping and portfolio completion and POCUS user status.

## Results

**Figure 1:**
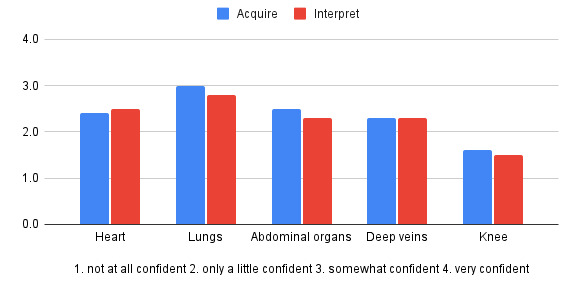
How confident are you in your ability to acquire and interpret the following views?

**Figure 2:**
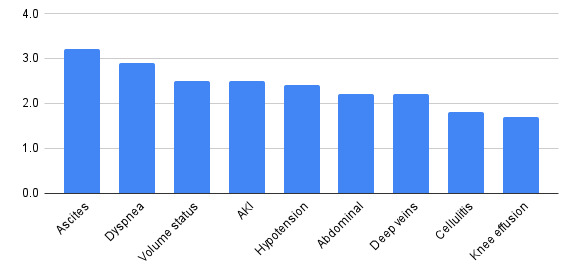
How likely are you to use ultrasound to evaluate the following?

**Figure 3:**
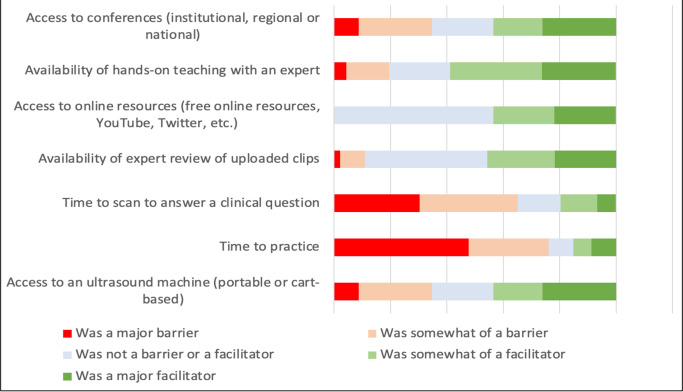
POCUS proficiency facilitators and barriers.

The survey achieved a response rate of 52% (46/89). Participant characteristics are shown in Table 1. The overall median knowledge test score was 72%, range 31-100%. Overall, graduates were most confident with image acquisition of the lung (Figure 1), and were most likely to use ultrasound to evaluate ascites and dyspnea (Figure 2). Time constraint was the most frequent barrier, whereas availability of an expert for hands-on teaching was the most important facilitator to developing proficiency in POCUS (Figure 3). Availability of an ultrasound machine was not a major barrier for the vast majority. Conferences, online resources and expert review of uploaded clips were all important facilitators to gain proficiency in POCUS.

**Table 1:** Participant characteristics at follow up.

Characteristic	All respondents n=46 (%)	Completed Portfolio n=11
Gender		
Male	23 (50)	8
Female	23 (50)	3
Practice Setting^		
Inpatient	37 (80)	10
Outpatient	15 (33)	4^^
Critical Care	2 (4)	1
Specialty		
General Medicine	41 (89)	10
Other Specialty	5 (11)	1
Portfolio Completion		
Yes	11 (24)	
Partial	10 (22)	
No	25 (54)	
Hours of POCUS education completed prior to course		
0	19 (41)	3
1-5	20 (43)	7
6-10	4 (9)	0
11-20	1 (2)	0
>20	2 (4)	1
Years in practice		
1-5	18 (39)	3
6-10	11 (24)	4
11-20	11 (24)	3
>20	6 (13)	1
Teaching setting		
UME, GME, or APP	37(80)	11
None	9 (20)	0
POCUS teaching currently		
Course leadership	4 (9)	4
Course teacher	6 (13)	3
Informal	20 (43)	4
None	16 (35)	0
I-ScaN cohort		
April 2018	9	5
March 2019	15	2
September 2020– May 2021	9	3
January–May 2022	7	0
February-June 2023	6	1

*Those self-identified as completing image portfolio

^More than one setting possible

^^ One does only outpatient catre

UME, Undergraduate Medical Education; GME, Graduate Medical Education, APP, Advanced Practice Provider

The characteristics of the 11 participants who reported completing the image portfolio are included in Table 1. All 11 reported actively teaching POCUS, whereas 54% of those who did not complete the portfolio were involved in teaching POCUS. Table 2 presents the median test scores of participants across two cohorts (2018-2019 and 2020-2023) based on their portfolio completion status and likelihood of POCUS use. Test results for participants in the earlier cohorts were not statistically different compared to those in the latter cohorts (median scores of 68% and 76%, respectively; p=0.35). We observed a significant difference in test scores between individuals who reported completing a POCUS portfolio and those who did not (median 92% and 68%, respectively; p <0.01) Users of POCUS, as defined by those reporting being somewhat or very likely to use POCUS to evaluate dyspnea, had significantly higher median test scores compared to non-users (78% and 65%, respectively; p<0.001). However, the interaction between cohort and POCUS use was not statistically significant. 

**Table 2:** Comparison of test score to portfolio completion and POCUS use by cohort year.

	**All cohorts (n=46)**	**P value **	**2018-2021** **cohorts (n=24)**	**2022-2023** **cohorts (n=22)**	**P Value **
	**Median test score (%)**		**Median test score (%)**	**Median test score (%)**	
All participants (n=46)	72		68	76	0.35
Portfolio completion					
Yes (n=11)	92	<0.001	96	88	0.056
No or partial (n=35)	68		64	72	
User vs non-user (defined as likelihood to use pocus for dyspnea as 3 or 4)					
User (n=29)	78	<0.001	84	80	0.9
Non-user (n=17)	65		66	64	

**Table 3:**
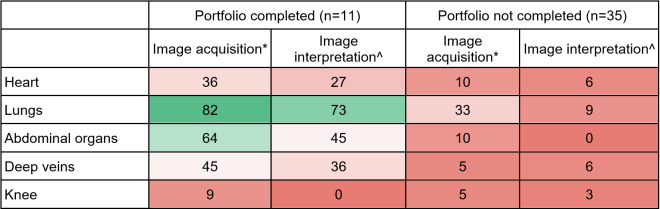
Confidence in image acquisition and interpretation by portfolio completion. Reported as the percentage choosing Very confident out of the 4-point Likert scale from Not very confident (1) to Very confident (4). Heat map reported as darker red to darker green for 0-100 range. *Aggregate Likert score of 3.2 for portfolio completers and 2.6 for non-completers (p<0.001). ^Aggregate Likert score of 3.0 for portfolio completers and 2.0 for non-completers (p<0.001)..

We found a significant difference in confidence levels between participants with a completed POCUS portfolio and those without, with aggregated Likert scores for image acquisition of 3.2 and 2.6, respectively (p < 0.001) and for image interpretation of 3.0 and 2.0, respectively (p < 0.001). Graduates who did not complete the portfolio reported low confidence across all systems, whereas the majority who did complete the portfolio were very confident about their lung and abdomen image acquisition skills (Table 3). Although confidence levels for cardiac ultrasound were not high even in the group of portfolio completers, there were increased confidence levels reported in completers as compared to the non-completers across all systems except for knee, which was not taught in this course. In the group of participants who completed a portfolio, only a single individual reported time constraints as a major barrier. In contrast, in the group who did not complete the portfolio, 60% reported time to practice as a major barrier (Table 4).

**Table 4:**
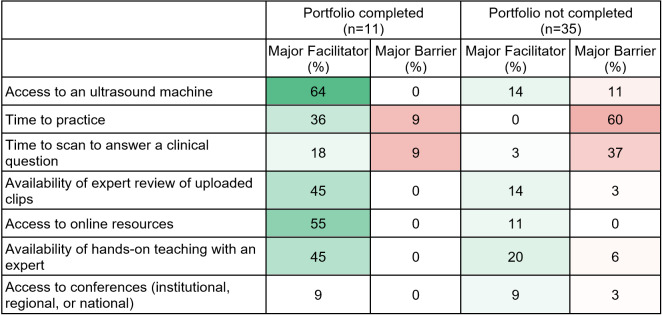
Facilitators and barriers by portfolio completion status. Reported as the percentage choosing Major Barrier or Major Facilitator out of the 5-point Likert scale from Major Barrier (1) to Major Facilitator (5). Heat map reported as darker green and darker red for higher percentage of Major Facilitator and Major Barrier, respectively.

## Discussion

In this analysis of faculty graduates of an internal medicine POCUS program at a single institution, we found that self-identified portfolio completion was associated with higher scores on a knowledge test, greater confidence in image acquisition and interpretation, and increased integration of POCUS into clinical practice. Those completing a portfolio were represented across inpatient and outpatient settings, years in practice, and were heavily represented as POCUS teaching faculty.

This study showed that self-identified completion of the image portfolio has a strong correlation to POCUS independence, as evidenced by high scores on the knowledge test, greater confidence in image acquisition and interpretation, and frequency of clinical use across a variety of clinical conditions. We believe that successful completion of a rigorous POCUS course followed by completion of a full image portfolio, alongside ongoing quality assurance, is strongly suggestive of clinical competency in POCUS.

Those in our cohort who completed the image portfolio reported major facilitators like having access to an ultrasound machine, online resources, and on-site experts. Programs intending to build a POCUS program will need to invest in these resources, though further barriers emerge even after training and machines are available 20. Time is commonly listed as a barrier to POCUS adoption, however, given the observational design of this study we are unable to determine why those completing a portfolio did not perceive time as a major limiting barrier. Though those faculty with protected or administrative time in their schedules might have more time to practice, it is our experience that most learning in POCUS is done during clinical rotations. Perhaps they were able to incorporate POCUS gradually in a manner that diminished time as a cost and were able to, as most independent users are, add POCUS as a time-saver. 

We have found that encouraging low-stakes teaching in POCUS offers a successful pathway for reliable development of POCUS independence. All of the 11 portfolio completers reported actively teaching POCUS, with 4 in a course leadership role, 3 as course faculty, and 4 teaching informally. Even the majority of portfolio non-completers (54%) reported teaching POCUS. As the adoption of POCUS at our institution has grown, students and residents are requesting POCUS education on the wards, fostering a culture that encourages our faculty to take our course, practice, and teach. Given that many faculty teachers already feel comfortable with routine bedside instruction, a highly effective strategy to teach POCUS is showing how ultrasound builds on a history and physical examination, as was described in the MGH experience. POCUS courses should dedicate resources to strategies favorable to maximizing independent practice such as protecting time for POCUS champions, enrolling learners who will practice and teach, providing guidance and support on longitudinal learning, and foster teaching in a variety of environments. Clinical integration of POCUS can be incorporated into existing teaching responsibilities faculty have with students, residents or APPs. This approach lowers the barrier for recent course graduates to teach students and residents who also have minimal POCUS experience. Pairing novice teachers with experienced teachers in POCUS courses or other educational settings has been an effective practice we routinely employ at our institution.

To our knowledge this is the only study to follow faculty participants of a POCUS program for up to five years after initial course completion. We did not identify an overall difference in test results based on time since completion, though the effect of portfolio completion over time approached statistical significance, supporting the educational theory that knowledge retention is improved through spaced learning and repetition. 

This study has a few limitations. The one-group post-test-only design is inherently subject to multiple threats to internal validity. Apart from the knowledge test, the survey relied on self-assessment of confidence and practice patterns, limiting the ability to draw conclusions regarding competency. Voluntary response bias may skew the results towards learners with a more favorable attitude towards POCUS, as evidenced by the disproportionate number of portfolio completers who responded to the survey. 

The results of this study may not be broadly generalizable across practices with variable access to resources, settings with fewer teaching expectations, and programs without current POCUS expertise to build from. Though the sample size of a pool of 89 participants is relatively small, it is representative of a diverse faculty pool at a large academic institution comprising multiple teaching hospitals. Although we were aiming for a higher response rate, 52% is comparable to other studies of its kind 14. Drawing conclusions about behaviors can be further accomplished through interviews employing theoretical models and frameworks 20. We believe there is value in further exploring adult learning theory as it relates to POCUS adoption. 

## Conclusions

In this long-term, single-institutional study of faculty graduates of an annual POCUS program, those having reported completing an image portfolio scored significantly higher on a knowledge test, reported higher confidence with image acquisition and interpretation, and reported using and teaching POCUS more frequently compared to graduates who did not complete the portfolio. Facilitating factors to achieving POCUS proficiency include access to ultrasound machines, online educational resources and on-site experts. Graduates should be encouraged to teach in low-stakes settings. POCUS programs should be designed to maximize completion of an image portfolio. 

Disclosure Statement 

Ethics approval and consent to participate: The I-ScaN program qualified as a quality improvement project by the NYU Grossman School of Medicine’s Institutional Review Board criteria using a self-certification process to ensure the data were not collected for research purposes. The NYU self-certification form that determined this project qualifies as quality improvement has been included in the submission. The primary goal of the project was to assess and improve teaching performance of the I-ScaN program. 

Informed Consent 

Participant consent was not obtained due to the qualification of this project as quality improvement (see attached NYU self-certification form).

## Funding Statement

This program was supported by a generous donation from the Goodman Family Foundation who did not participate in any aspect of this project, including study design, collection, analysis or data interpretation, or in writing of the manuscript.

## Declaration of Conflict of Interest

The authors declare no conflict of interest.

## Supplementary Materials

Supplementary Appendix S1 ISCAN combined Knowledge Test and Survey.

Supplementary Appendix S2 Comparison and description of test questions administered in 2018 and in 2019-2023.
