# Supplementary material for: Knowledge and Confidence Among Five Cohorts of Faculty Learners in a Point of Care Ultrasound (POCUS) Program: Factors Defining Learner Success
Source: POCUS J. 2024 Nov 15;9(2):109–16. doi: 10.24908/pocus.v9i2.17640 (PMC11616799; doi:10.24908/pocus.v9i2.17640)
Supplement: Supplementary Appendix S1 [file pocusj-09-02-17640-s001.pdf]

ISCAN combined Knowledge Test and Survey

Start of Block: ISCAN survey

Q1 intro A link to the answer key will be included at the conclusion of the survey.

Q2 acquire How confident are you in your ability to **ACQUIRE** ultrasound images of the:

	Not at all confident (1)	Only a little confident (2)	Somewhat confident (3)	Very confident (4)
Heart (1)	○	○	○	○
Lungs (2)	○	○	○	○
Abdominal organs (3)	○	○	○	○
Deep Veins (4)	○	○	○	○
Knee (5)	○	○	○	○

Q3 interpret How confident are you in your ability to **INTERPRET** ultrasound images of the:

	Not at all confident (1)	Only a little confident (2)	Somewhat confident (3)	Very confident (4)
Heart (1)	○	○	○	○
Lungs (2)	○	○	○	○
Abdominal organs (3)	○	○	○	○
Deep Veins (4)	○	○	○	○
Knee (5)	○	○	○	○

Q4 evaluate How likely are you to use US to evaluate:

	Not at all likely (1)	Only a little likely (2)	Somewhat likely (3)	Very likely (4)
Dyspnea (1)	o	o	o	o
Abdominal pain (2)	o	o	o	o
Hypotension (3)	o	o	o	o
Volume status (4)	o	o	o	o
DVT (5)	o	o	o	o
Celluitis (6)	o	o	o	o
Knee infusion (7)	o	o	o	o
Ascites (8)	o	o	o	o
Acute kidney injury (9)	o	o	o	o

Q5 procedure Click to write the question text

	All procedures (1)	Only for challenging procedures (2)	I don't perform this procedure (3)	I don't use US for this procedure (4)
Venipuncture (1)	○	○	○	○
Lumbar puncture (2)	○	○	○	○
Paracentesis (3)	○	○	○	○
Peripheral IV (4)	○	○	○	○
Thoracentesis (5)	○	○	○	○

Q6 proficiency Have you reached the level of proficiency with POCUS you have hoped to since taking the course?

○ No (1)

○ Partly (2)

○ Yes (3)

Q7 influence What influenced your proficiency in learning POCUS?

	Was a major barrier (1)	Was somewhat of a barrier (2)	Was not a barrier or facilitator (3)	Was somewhat of a facilitator (4)	Was a major facilitator (5)
Access to an ultrasound machine (portable or cart-based) (1)	o	o	o	o	o
Time to practice (2)	o	o	o	o	o
Time to scan to answer a clinical question (3)	o	o	o	o	o
Availability of expert review of uploaded clips (4)	o	o	o	o	o
Access to online resources (free online resources, YouTube, Twitter, etc.) (5)	o	o	o	o	o
Availability of hands-on teaching on-sight with an expert (6)	o	o	o	o	o
Access to conferences (institutional, regional, or national) (7)	o	o	o	o	o

Q8 interview We are interested in better understanding what contributes to clinicians' decisions about whether or not to use POCUS. Would you be willing to be interviewed individually or in a focus group?

- No (1)
- Yes (2)

Q9 thoughts Please share any additional thoughts you have about POCUS or its use.

____

Q10 gender Gender

- Male (1)
- Female (2)
- Non-binary / third gender (3)
- Prefer not to say (4)

Q11 age Age

- 20-30 (1)
- 31-40 (2)
- 41-50 (3)
- 51-60 (4)
- >60 (5)

Q12 year In what year did you take the NYU POCUS course? (Choose the first course if multiple)

- 2018 (1)
- 2019 (2)
- 2020 (3)
- 2021 (4)
- 2022 (5)
- 2023 (6)

Q13 porftolio Have you completed an image portfolio?

- No (1)
- Partially completed (2)
- Yes (3)

Q14 courses How many hours of additional formal POCUS education have you received outside of this course? Include electives, POCUS Refresher, ACP course, etc.

- 0 (1)
- 1-5 (2)
- 6-10 (3)
- 11-20 (4)
- >20 (5)

Q15 degree Degree

- ○ MD/DO (attending) (1)
- ○ PA (2)
- ○ NP (3)
- ○ Other - indicate below (4) ____

Q16 sepcialty What is your specialty? (general medicine, nephrology, neuro critical care, etc.)

____

Q17 years practice How many years have you been practicing in your current field?

____

Q18 practice setting Practice setting (choose all that apply)

- ▢ Inpatient (1)
- ▢ Outpatient (2)
- ▢ Critical care (3)

Q19 teach pocus Do you teach POCUS to others? (choose all that apply)

- ☐ Informally, as I would teach other clinical skills (1)
- ☐ Dedicated POCUS teaching as part of a larger course (2)
- ☐ POCUS course leadership (3)
- ☐ I do not teach POCUS (4)

Q20 learners Who are your learners (POCUS and non-POCUS related)? (choose all that apply)

- ☐ Faculty (1)
- ☐ Medical Students (2)
- ☐ Residents (3)
- ☐ Fellows (4)
- ☐ APPs (PAs, NPs) (5)
- ☐ I don't teach (6)

End of Block: ISCAN survey
